# One-Pot Mechanochemical Synthesis of Carbons with High Microporosity and Ordered Mesopores for CO_2_ Uptake at Ambient Conditions

**DOI:** 10.3390/nano13152262

**Published:** 2023-08-07

**Authors:** Rabindra Dubadi, Mietek Jaroniec

**Affiliations:** Department of Chemistry and Biochemistry, Kent State University, Kent, OH 44242, USA; rdubadi@kent.edu

**Keywords:** mechanochemical synthesis, self-assembly, block copolymer templating, CO_2_ uptake

## Abstract

Mechanochemical synthesis of ordered mesoporous carbons with tunable mesopores and well-developed irregular microporosity is investigated. This synthesis was carried out by the self-assembly of ecofriendly chemicals such as tannin and glyoxal used as carbon precursors, and triblock copolymer as a soft templating agent. The structural properties of the resulting carbons were tailored by using different block copolymers (Pluronic F127, and P123) as soft templates. The various weight ratios of tannin and block copolymer were employed to tune the textural properties of these carbons. The tannin: Pluronic F127 ratios (1:0.75, 1:1, 1:1.1) gave the ordered mesoporous carbons among a wide variety of the samples studied. The ordered mesoporosity was not observed in the case of Pluronic P123 templated mesoporous carbons. The CO_2_-activated carbon samples obtained for both Pluronic templates showed a high specific surface area (close to 900 m^2^/g), large pore volume (about 0.6–0.7 cm^3^g^−1^), narrow pore size distribution, and high CO_2_ uptake of about 3.0 mmol g^−1^ at 1 bar pressure and ambient temperature.

## 1. Introduction

The growing interest in the design and development of nanoporous carbon materials is observed due to their unique and wide applications such as adsorption, energy storage/conversion, drug delivery, and catalysis. These materials continue to attract attention among chemists, physicists, and material science researchers. There are different types of carbon materials such as carbon nanotubes, graphene, and porous carbons. The latter attracted a significant attention because of their usage in adsorption, catalysis, energy, and environmental applications [1]. Moreover, the synthesis of porous carbons is simple and allows for tunning their structural and surface properties [2]. IUPAC defines porous materials based on their pore sizes as microporous (<2 nm), mesoporous (2–50 nm), and macroporous (>50 nm). Further, the micropores having pore sizes (<0.7 nm) are defined as ultramicropores [3]. The ability of synthesizing highly porous carbons with micro-, meso- and macropores make these materials applicable in different fields [4,5,6,7]. While microporosity in carbons can be created by carbonization of oxygen rich carbon precursors or by chemical and/or physical activation, their mesoporosity can be developed by hard and/or soft templating strategies [8]. In the hard templating strategy, the mesopores of hard templates (usually mesoporous silicas) are filled with carbon precursors via infiltration or polymerization, followed by carbonization and template removal. This multi-step strategy involves toxic/harsh chemicals, and is time consuming and costly, which limits its applications [9]. On the other hand, the soft templating strategy is easy, safe, and faster [10]. In this process safer carbon precursors can be used such as different types of biomasses [11,12,13,14] to obtain the desired porous carbons and make the procedure greener. Usually, the soft templating strategy is performed in a liquid phase, preferably aqueous phase. Hence, the development of new solvent-free, eco-friendly, safe, and easy routes for the synthesis of porous carbons is a highly desirable goal, which could be accomplished by mechanochemical strategy [15,16,17,18]. For instance, this strategy has been used to fabricate carbons with tunable and ordered mesopores [19,20].

Synthesis of ordered mesoporous materials has been greatly advanced after the discovery of ordered mesoporous silica in 1992 by Mobil oil company [21]. Especially, ordered mesoporous carbons (OMCs) found numerous applications in different fields such as adsorption, separation, and catalysis. OMCs have often been synthesized by using hard templates such as SBA-15 [22], SBA-16 [23], and FDU-12 [24]. Moreover, the soft templating approach has been used for the preparation of OMCs [25]. Braghiroli et al. synthesized OMCs using the self-assembly of mimosa tannin with a micellar solution of Pluronic F127 as a soft template. During this process 12 N HCl was used to adjust the pH of the system to form tannin polymer [10]. This ecofriendly solvent-based method was further extended by Phuriragpitikhon et al. without the use of acid to obtain microporous–mesoporous carbons [26]. Potassium salt as well as CO_2_ activation was also used to form highly microporous–mesoporous carbons. This study simplifies the solvent-based method developed by Phuriragpitikhon et al. by employing the mechanochemical synthesis with a very small volume of water and ethanol. Mechanochemical synthesis is the most effective eco-friendly alternative to the conventional solvent-based method for the preparation of porous materials [27,28]. Due to the recent advancements and popularity of this method, in 2003 IUPAC defined a mechanochemical reaction as “a chemical reaction that is induced by the direct absorption of mechanical energy” [29]. Mechanochemical method involves transformation of reactants to the product using mechanical forces such as compression, fractures, shear, or friction [15,30,31]. Mechanochemistry has already been used for the synthesis of various materials such as alumina [32], metal oxides [33], and carbon [34]. Furthermore, Zhang et al. used mechanochemical way to synthesize OMC using the self-assembly of tannin and Pluronic F127. They used two step synthetic procedure at 1200 rpm to incorporate a metal salt followed by the rigorous stepwise N_2_ carbonization to obtain OMC [35]. In another study highly ordered porous sp^2^ carbon at room temperature was synthesized mechanochemically using organic chemical and inorganic salt [17]. Similar synthesis of OMC was carried out by Castro-Gutiérrez et al. by using one step mechanochemical processing in the presence HCl to adjust pH followed by carbonization under N_2_ and then physical activation with CO_2_. Similarly, the chemical activation was also investigated using KOH and CH_3_CO_2_K as an activator [36]. Here OMCs are prepared using one step mechanochemical processing of a green precursor, tannin, followed by one step CO_2_ activation without carbonization and without using any toxic chemicals. In this study we adopted green synthesis (mechanochemistry) to challenge the wet chemical methods to prepare highly porous carbons. Many existing green recipes involve multi-step processing and employ one or more solvents and toxic chemicals. To avoid the use of solvents and toxic chemicals, and reduce the cost and time of synthesis, we modified/replaced the existing recipes by mechanochemical processing, which is simple, fast, and can be performed without solvents or with a very small volume of green solvents such as water and ethanol to assist ball milling. In addition, we eliminated and/or replaced toxic chemicals in the mechanochemical synthesis of highly porous carbons by using nontoxic or low toxicity ones. Additionally, we used two different Pluronic triblock copolymers (F127, P123), which are non-toxic, inexpensive, and commercially available to investigate the textural properties of the resulting carbons. Furthermore, the role of nontoxic aldehyde, glyoxal as a cross linking agent was also investigated. This ecofriendly method affords carbon materials with large amount of small micropores essential for CO_2_ uptake at ambient conditions, which in addition possess the well-developed mesoporosity that facilitates adsorption kinetics.

## 2. Materials and Methods

### 2.1. Materials

All chemicals were used as received without further purification. Commercial Mimosa tannin, used as a biomass-type carbon precursor was provided by Silva-Chimica. Poly (ethylene oxide)-poly (propylene oxide)-poly (ethylene oxide) triblock copolymers, Pluronic P123 and F127, were provided by BASF. Glyoxal (pure, 40 wt% solution in water) was purchased from Acros Organics, and ethanol (200 Proof) was supplied by Sigma-Aldrich, St. Louis, MO, USA. Deionized water purified by the Milli-Q water purification system was used during ball milling.

### 2.2. Synthesis of Microporous–Mesoporous Carbons

**Figure 1:**
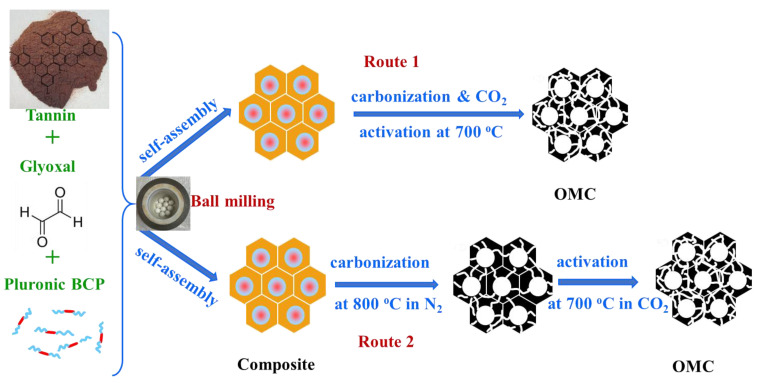
Schematic illustration of the mechanochemical synthesis of OMCs using tannin, glyoxal, and block copolymer subjected to one-step carbonization and CO_2_ activation (route 1) and carbonization under N_2_ and subsequent CO_2_ activation (route 2).

An amount of 2 g of Mimosa tannin and an equal amount of Pluronic F127 (Tannin: Polymer ratio of 1:1) as a soft template were added with 1 mL of deionized water (DI) and 3 mL of 200 Proof ethanol. Then, 2 mL of glyoxal (40 wt% solution in water) was added and milled for 2 h at 500 rpm using Planetary ball mill (PM200, Retsch, Hann, Germany) in an yttria-stabilized zirconia (YZrO_2_) grinding jar equipped with eight yttria-stabilized ZrO_2_ balls, 1 cm in diameter each. The obtained brownish viscous mixture was transferred to a petri-dish and dried at 100 °C for a couple of hours to remove the solvent. The dried polymer composite was then subjected to a simultaneous carbonization-CO_2_ activation at the heating rate of 1 °C/min up to 700 °C for 3 h under flowing CO_2_ to obtain ordered mesoporous carbon. For a comparison, porous carbon samples were prepared by carbonization in N_2_ at 800 °C for 2 h and then activated under CO_2_ at 700 °C for 3 h as shown in Figure 1. Similarly, the control sample was synthesized without using the cross-linking agent, i.e., glyoxal. The carbon sample obtained by using only Mimosa tannin (T) was also studied as a reference. On the other hand, the other porous carbon samples were prepared by varying the tannin/polymer weight ratios. To further investigate the nature of the carbon samples, we changed the soft template to P123 and prepared some samples according to the above-mentioned method. The change in hydrophilic chain length in P123 might affect the self-assembly process and hence the textural properties of the obtained sample can be different. The obtained samples were named TFG-X and TPG-X, where symbols with F and P refer to the carbon samples templated with Pluronic F127 and P123, respectively, T refers to Mimosa tannin, G refers to glyoxal, and X denotes the tannin/ polymer weight ratio. Table 1 provides the list of the samples studied together with their notation and carbon yield.

### 2.3. Measurements and Characterization of Microporous–Mesoporous Carbons

Nitrogen adsorption–desorption isotherms were conducted at −196 °C. Carbon dioxide adsorption isotherms were measured at 25 °C. Each sample was degassed under vacuum for 2 h at 200 °C prior to the N_2_ and CO_2_ adsorption measurements. Nitrogen adsorption measurement for TFG-1 was repeated to obtain sets of two adsorption isotherms confirming reproducibility of the adsorption data. Analysis of adsorption data is presented in the results and discussion section. The possible errors of the adsorption/desorption measurements on the commercial analyzer instruments are discussed somewhere [37]. In addition, powder X-ray diffraction (XRD) analysis was performed on Rigaku Miniflex 600 X-ray diffractometer using Cu Ka radiation operating at current, voltage, and scan rate of 15 mA, 40 kV, and 0.020°, respectively. The structural information (porosity) was investigated using high resolution transmission electron microscopy (TEM). The TEM images were obtained using a FEI Tecnai F20ST/STEM instrument operated at 200 kV. The samples were loaded on the carbon coated copper grids by dipping during TEM study. Thermogravimetric analysis was obtained by using a TGA Q500 thermogravimetric analyzer using a high-resolution mode in flowing nitrogen with a heating rate of 10 °C/min up to 800 °C. The elemental analysis (CHN) was performed, and the results are presented in Table 2. This analysis indicates that the carbon sample contains mainly carbon (>80%). The elemental analysis was conducted on a LECO TruSpec Micro CHNS instrument.

### 2.4. Calculations

The specific surface area (S_BET_) of the synthesized carbon samples was calculated from nitrogen adsorption isotherm data using the Brunauer–Emmett–Teller (BET) method in the relative pressure (P/P_0_) range of 0.05–0.2 [38]. The total pore volume (V_T_) was determined using the volume of adsorbed N_2_ at 0.98 P/P_0_. The presence of mesopores, in addition to TEM imaging, was confirmed by the H2 adsorption hysteresis loop. Pore size distribution (PSD) curves in the mesopore range were determined using the Kruk–Jaroniec-Sayari (KJS) method [39]. However, PSDs in the micropore region were determined using 2D-nonlocal density functional theory (2D-NLDFT) [40]. The volume of micropores (V_mi_ < 2 nm, V < 1 nm, and V < 0.7 nm) were obtained from the cumulative pore volume distribution. Then the following equation was used to determine the mesopore volume (2–50 nm) for the samples studied.

V_me_ = V_T_ − V_mi_ (1)

### 2.5. CO_2_ Activation

Usually, preparation of porous carbons is a two-step process including (i) carbonization of a carbon precursor to produce biochar and (ii) activation using CO_2_ or steam. Activation process is used to enlarge microporosity in carbonized or non-carbonized samples [41]. The main reactions involved in physical activation are given below [6].

C + H_2_O → CO + H (2)

2C + H_2_ → 2C_x_H_y_ (3)

C + CO_2_ → 2CO (4)

CO + H_2_O → CO_2_ (5)

The physical activation with CO_2_ follows reaction 4 and enlarges the surface area and micropore volume.

The elemental analysis of the synthesized samples (Table 2) shows mostly carbon (>80%), a small amount of hydrogen, and the remaining percentage refers to oxygen. The main flavonoid unit in mimosa tannin has about 60% carbon; however, this synthetic route after carbonization process results in higher yield of carbon in the final product. This high carbon yield is beneficial for scaling up the production of carbon materials.

## 3. Results and Discussion

### 3.1. Thermogravimetric Studies

**Figure 2:**
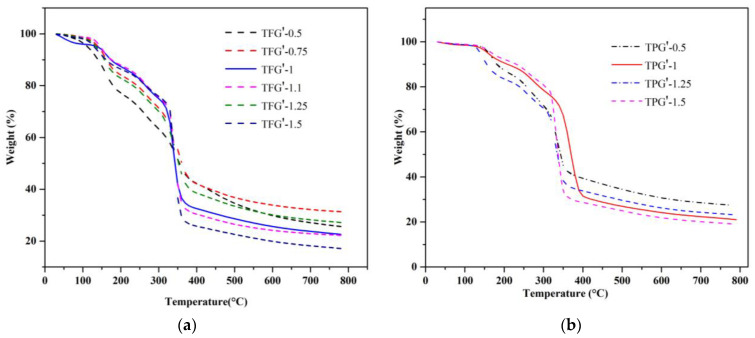
TGA profiles of TFG′ tannin–polymer composite samples with different (T/F) weight ratios (**a**), and TPG′ samples with different (T/P) weight ratios (**b**), where apostrophe symbol refers to the TFG and TPG tannin–polymer composite samples (before carbonization).

Thermal stability of porous carbons obtained through one step mechanochemical synthesis was investigated by thermogravimetric analysis in the flowing N_2_. The TG profiles for the selected samples were collected from 30 to 800 °C at the rate of 10 °C/min. Figure 2. shows the TG profiles for both F127 and P123-templated tannin–polymer composite samples. The initial part of the TG profiles up to 120 °C indicates the weight change due to the removal of adsorbed water. The second weight change between 120 and about 300 °C indicates the formation of some gaseous products such as CO_2_ and H_2_. Similarly, the decomposition of Pluronic, and the cross linker (i.e., glyoxal) is indicated by the third weight loss at around 400 °C [26,42,43]. The carbonized samples are carbon-rich (>80%) as indicated by the residue at about 800 °C (see Table 2).

### 3.2. TEM Imaging of Mesostructures

**Figure 3:**
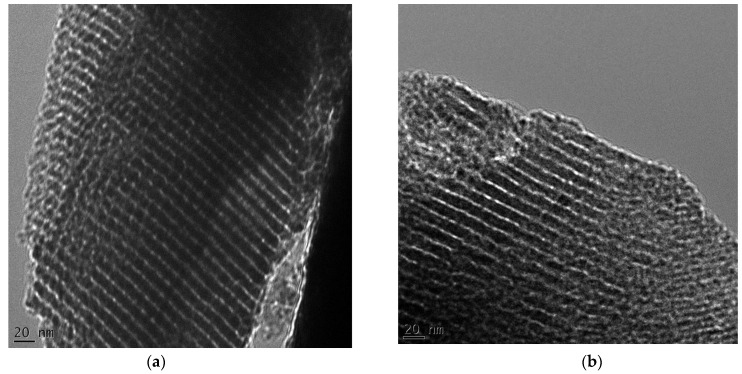
TEM images of TFG-1 (CO_2_ activated) (**a**), and TFG-1 (nonactivated) (**b**).

**Figure 4:**
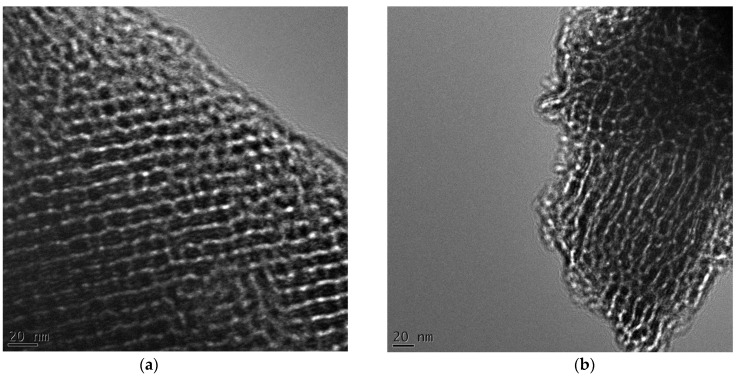
TEM images of TFG-0.75 (**a**), and TFG-1.1 (**b**).

The structural properties of the prepared carbon samples were analyzed by transmission electron microscopy (TEM). The synthesized samples with tannin/polymer (F127) (T/F) weight ratio of (1:1) show ordered mesoporosity. Figure 3a shows ordered mesoporous carbon obtained through a single-step CO_2_ activation. Similar structured mesoporous carbon was obtained for non-activated samples (see Figure 3b). Analogously, the carbon samples prepared by using (T/F) weight ratios of 1:0.75, and 1:1.1 also possess ordered mesopores (see Figure 4a,b). The carbon samples templated with P123 were also investigated but in this case mesopores are uniform but disordered (see Figure S1). Furthermore, the mesostructures of the carbon samples (TFG-X, X = 0.5, 1.25, and 1.5) were also investigated. These samples have disordered mesopores as shown in Figure S2.

**Figure 5:**
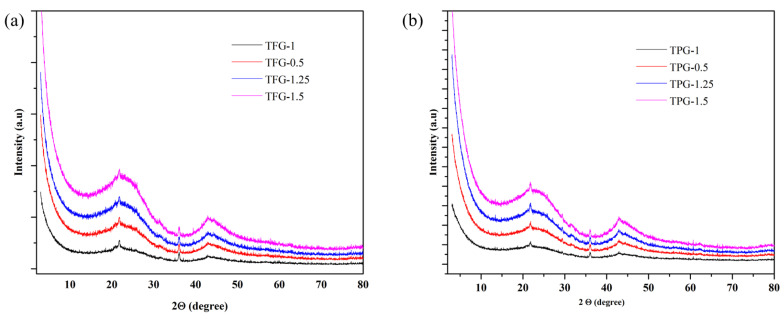
XRD spectra of TFG carbon samples with different (T/F) weight ratios (**a**), TPG carbons with different (T/P) weight ratios (**b**).

The wide-angle X-ray diffraction (XRD) patterns in Figure 5a,b refer to the amorphous nature of carbons as evidenced by broad low intensity peaks around 23 and 44° (2Θ degrees) [44].

### 3.3. Nitrogen Adsorption Studies

**Figure 6:**
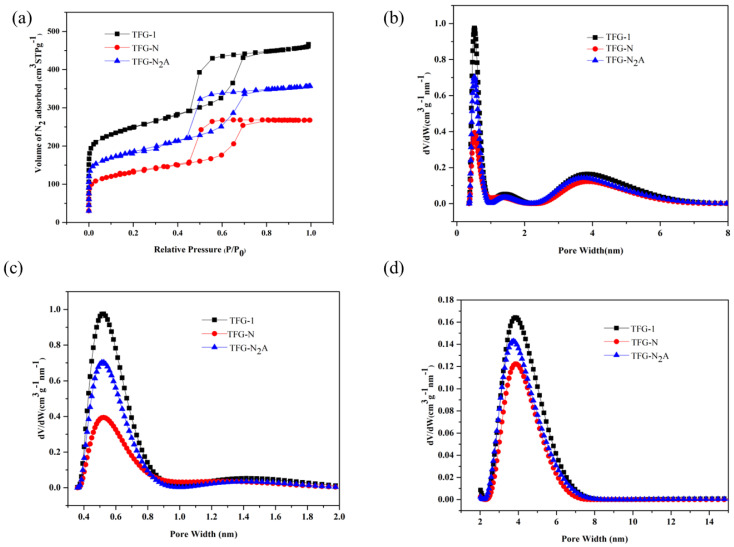
N_2_ adsorption isotherms for ordered mesoporous carbon TFG-1 samples with and without activation (**a**). The pore size distribution in the range of micro and mesopores is displayed in panel (**b**), while panels (**c**,**d**) show pore size distributions in the range of micropores and mesopores, respectively.

Information about textural properties of the synthesized carbon samples was obtained from the N_2_ adsorption isotherms. The shape of the adsorption isotherms, which are type IV with H2 hysteresis loop, is characteristic for mesoporous materials [3]. The adsorption isotherms for TFG-1 carbon samples with and without activation are shown in Figure 6a. The steep capillary condensation steps on the isotherms further confirm the presence of uniformly distributed ordered mesopores in the carbon samples as depicted in TEM images on Figure 3. The pore size distributions (PSDs) in various pore ranges are depicted in Figure 6b–d. The adsorption isotherm and the respective PSD for the repeated sample for T/F (1:1) are shown in Figure S3. Similarly, the adsorption isotherms and their respective PSDs for ordered mesoporous carbons for T/F (1:0.75, and 1:1.1) are shown in Figure S4. On the other hand, the disordered mesopores shown in TEM images (see Figure S2) for different T/F weight ratios used to prepare TFG carbons are further proved by adsorption isotherms and their respective PSDs as shown in Figure S5.

**Figure 7:**
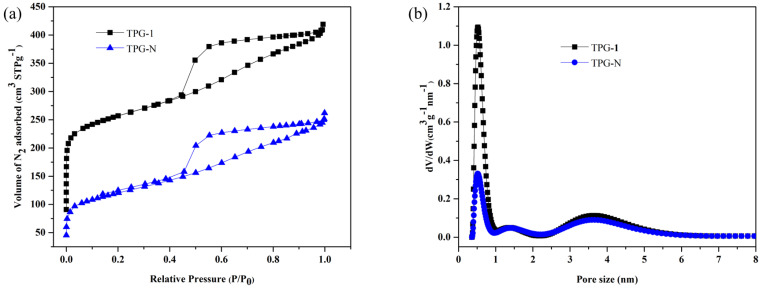
N_2_ adsorption isotherms for TPG-1 with and without activation (**a**) and the corresponding pore size distributions (**b**).

On the other hand, the adsorption isotherms for the TPG-1 carbon samples and the corresponding PSDs are displayed in Figure 7. These isotherms also show the presence of mesopores, and the PSDs further confirm the presence of micropores and ultramicropores. However, the shape of adsorption isotherms indicates the presence of uniform but disordered mesopores (see Figure S1). Additionally, the P123-templated carbon samples obtained for other weight ratios of T/P show similar adsorption isotherms (see Figure S6). Similarly, the textural properties of the reference carbons are summarized in Table S1. The advantage of glyoxal as a cross linking agent is manifested by the improved textural properties of the synthesized carbons as evidenced by the enhanced surface area and pore volume values. On the other hand, the reduced surface area, and lower pore volume is observed for the samples prepared without glyoxal (Table S1). This means, glyoxal supports the self-assembly of tannin during ball milling.

The textural properties of the carbon samples studied are shown in Table 3. The values in Table 3 indicate the advantage of one step CO_2_ activation and mechanochemical processing. In comparison to the solvent-based method used by Phuriragpitikhon et al. [26], this study shows the comparable results in terms of pore volumes without using potassium oxalate as an in situ activator. The mechanochemical synthesis used in this work affords carbons with higher total pore volume (0.72 cm^3^g^−1^; increase by ~76%) for TFG-1 (one step carbonization and activation in CO_2_) vs. 0.41 cm^3^g^−1^ for TFG-1N sample (no CO_2_ activation). This ample enlargement in the total pore volume was also achieved for the CO_2_ activated P123 templated carbon (0.63 vs. 0.38 cm^3^g^−1^). However, for the F127 templated carbon this enhancement is higher. On the other hand, the volume of ultramicropores is larger in the case of P123 templated carbon samples, which leads to higher CO_2_ uptake.

### 3.4. Carbon Dioxide Adsorption

**Figure 8:**
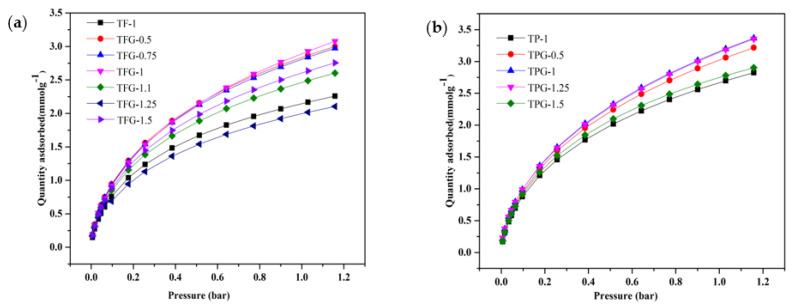
CO_2_ capture by TFG-1 carbons obtained with different (T/F) weight ratios (**a**), and TPG-1 samples with different (T/P) weight ratios of tannin to polymer (**b**).

The CO_2_ adsorption isotherms for mechanochemically synthesized carbon samples are measured at 25 °C and 1.03 bar pressure are shown in Figure 8a,b and the amount of CO_2_ captured is listed in Table 3. N-doped ordered mesoporous carbon spheres synthesized by Chen et al. and tested for CO_2_ capture adsorbed 2.43 mmolg^−1^ of CO_2_ at 25 °C and ~1 atm [20], which is lower than the value reported in this study. Adsorption of CO_2_ in highly porous carbons is physical in nature, i.e., occurs via Van der Waals type of interactions [45]. The presence of both ultramicropores and small mesopores are beneficial for the CO_2_ capture [46]. According to the textural properties values given in Table 3, the volume of ultramicropores is higher in P123 templated samples with larger mesopore volume. Hence, the availability of suitable amount of micro and mesopores favors higher CO_2_ capture at ambient conditions [46]. In the case of TPG-1 sample, about 48% (0.25 cm^3^g^−1^) of the total pore volume is contributed by ultramicropores and it has about 10% of micropores between 0.7 and 1 nm. These values are beneficial for CO_2_ adsorption in ambient conditions without any in situ chemical activation or functionalization. It is clear from Table 3 that the volumes of ultramicropores are lower for F127 templated carbon samples and hence they show smaller CO_2_ adsorption under similar conditions. It is well known [47] that ultramicropores are essential for high CO_2_ uptake at ambient conditions because in such pores physical adsorption of CO_2_ is enhanced due to the overlapping adsorption forces from the opposite pore walls.

The microporous–mesoporous carbons derived from mimosa tannin via mechanochemical synthesis and carbonization show a notable CO_2_ capacity due to larger volume of micropores below 1 nm. Table 3 demonstrates that the microporous–mesoporous carbon synthesized mechanochemically exhibits higher specific surface area and large volume of fine micropores, which are essential for high CO_2_ adsorption capacity that is comparable to or even better than those reported elsewhere. It is important to note that this study underscores the advantages of employing milder chemicals without using any activating agents, reduced amount of solvent, cleaner washing process, and adopting a more environmentally friendly production process that can be easily scaled up as indicated by high percentage yield of the product (see Table 1).

In comparison to similar previous studies (see Table 4), the amount of CO_2_ captured at ambient conditions is not only comparable but even better for carbons obtained by this ecofriendly one step mechanochemical process. While many previous studies employed a combination of physical and chemical activation methods, our approach took a different path. We opted for a one-step direct CO_2_ activation process, completely bypassing the need for chemical activation. Surprisingly, this unconventional method resulted in an even higher CO_2_ uptake, surpassing the expectations set by the conventional approaches.

These exceptional findings serve as an encouragement for modification of the existing activation processes commonly employed in the processing of carbons for CO_2_ capture applications.

## 4. Conclusions

The study presents an improved one-pot mechanochemical synthesis of highly microporous and ordered mesoporous carbons by using Mimosa tannin, glyoxal, and a triblock copolymer. This method simplifies the previous preparations and eliminates the need for using toxic chemicals for polycondensation of phenol formaldehyde resin adopted in wet chemistry. The one-step CO_2_ activation process creates a large volume of micropores, resulting in an increased surface area while significantly shortening the activation process. This study reveals that by varying the weight ratio of tannin to polymer (F127) may result in ordered and disordered mesoporosity; ordered carbon mesostructures were obtained for the weight ratios (T/F) of tannin to F127 polymer equal to 1:0.75, 1:1, and 1:1.1, while disordered carbon mesostructures were observed beyond the above-mentioned range, e.g., for 1:1.25 T/F ratio. However, for P123 polymer only disordered carbon mesostructures were produced. It is worth mentioning that for 1:1 and 1:1.25 weight ratios of tannin to polymer (T/P) for both F127 and P123 polymers the resulting mesoporous carbons showed the highest S_BET_ surface area, total pore volume, and micropore volume (see Table 3). The porous carbons studied here exhibited higher CO_2_ uptake at ambient temperature and pressure than the previously reported tannin-derived porous carbons. Thus, the mechanochemical synthesis of porous carbons that uses condensed tannins (biomass), and less toxic aldehydes offers a safer, easier, less expensive, and more environmentally friendly alternative for the development of carbons with tailored porosity. In this study the structural advantages are clearly presented as the carbon samples possess large volumes of ultramicropores, which assure high CO_2_ uptakes at ambient conditions, while the well-developed microporosity improves adsorption kinetics. The synthesized samples have a potential for practical environmental applications due to their high CO_2_ uptake at ambient conditions and can be used for various environmental, energy, and bio-related applications.

This eco-friendly method employed in our study not only affords porous carbon materials but also ensures their unique properties that are highly advantageous for CO_2_ uptake at ambient conditions. By utilizing this approach, we were able to achieve carbon materials with a significantly increased amount of small micropores, which are essential for efficient CO_2_ capture. These small micropores offer a multitude of benefits, primarily due to their high surface area and selective adsorption capabilities. They provide an optimal environment for CO_2_ molecules to be attracted and trapped, resulting in a higher uptake capacity for CO_2_ in ambient conditions.

## Figures and Tables

**Table 1:** Basic information about the carbon samples studied.

Sample Notation	Tannin/Polymer wt. Ratio	Carbon Yield (g)	% Yield
TFG-0.5	1:0.5	1.22	61.2
TFG-0.75	1:0.75	1.04	51.9
TFG-1	1:1	1.16	58.1
TFG-1.1	1:1.1	1.07	53.3
TFG-1.25	1:1.25	1.22	61.1
TFG-1.5	1:1.5	0.98	49.0
TPG-0.5	1:0.5	0.91	45.6
TPG-1	1:1	0.91	45.5
TPG-1.25	1:1.25	0.86	43.0
TPG-1.5	1:1.5	0.94	46.7
TF-1	1:1	1.01	50.3
TP-1	1:1	0.80	40.2
T	-	0.49	24.5

Notation: T—Mimosa Tannin, F—Pluronic F127, P—Pluronic P123, G—Glyoxal, the number at the end of the sample code indicates the tannin/polymer weight ratio.

**Table 2:** Elemental analysis data for the selected carbon samples.

Symbol	Carbon (%)	Hydrogen (%)
TFG-0.5	84.97	0.99
TFG-0.75	87.50	1.16
TFG-1N	84.63	0.66
TFG-1.1	85.50	0.89
TFG-1.25	85.39	0.78
TFG-1.5	83.80	0.79
TPG-0.5	84.98	0.75
TPG-1N	85.71	1.17
TPG-1.25	81.13	0.61
TPG-1.5	85.74	0.68
TF-1	84.59	0.94
TP-1	87.18	0.83
T	77.30	0.49

Note: TFG-X and TPG-X refer to the carbon samples prepared by using Mimosa tannin with Pluronic F127 and P123, respectively, along with glyoxal as a cross linking agent, X denotes the tannin/polymer weight ratio, while N refers to the carbon samples carbonized in nitrogen without CO_2_ activation.

**Table 3:** The textural properties of microporous–mesoporous carbons studied.

Sample	V_T_ (cm^3^g^−1^)	V < 0.7 nm (cm^3^g^−1^)	V < 1 nm (cm^3^g^−1^)	V < 2 nm (cm^3^g^−1^)	V_me_ (cm^3^g^−1^)	Micropore Width at PSD Max, w (nm)	S_BET_ (m^2^g^−1^)	n_CO2_ (25 °C) (mmolg^−1^)
TFG-0.5	0.59	0.16	0.19	0.22	0.37	0.55	663	2.87
TFG-0.75	0.54	0.16	0.18	0.21	0.33	0.78	766	2.84
TFG-1	0.72	0.22	0.25	0.29	0.43	0.52	868	2.93
TFG-1.1	0.58	0.22	0.25	0.28	0.30	0.78	634	2.49
TFG-1.25	0.73	0.22	0.26	0.30	0.43	0.76	883	2.01
TFG-1.5	0.63	0.19	0.21	0.25	0.28	0.56	610	2.64
TPG-1	0.63	0.25	0.30	0.33	0.30	0.52	888	3.20
TPG-0.5	0.59	0.16	0.19	0.22	0.37	0.55	663	3.06
TPG-1.25	0.73	0.22	0.26	0.30	0.43	0.76	883	3.18
TPG-1.5	0.63	0.19	0.21	0.25	0.28	0.56	610	2.78

Note: TFG and TPG refer to the carbon samples prepared by using Mimosa tannin and glyoxal as a cross linking agent with Pluronic F127 and P123, respectively; S_BET_-specific surface area calculated using the BET equation for adsorption data in the relative pressure range of 0.05–0.20; Single point pore volume obtained from the volume adsorbed at 0.98 P/P_0_; Pore diameter at the maximum of PSD obtained by NLDFT for micropores; n_CO2_–amount of CO_2_ adsorbed at 1.03 bar.

**Table 4:** Comparison of the CO_2_ uptake on the carbons studied with data reported in the literature on various porous carbons at ambient conditions.

Sample	Activator	S_BET_ (m^2^g^−1^)	V_T_ (cm^3^g^−1^)	CO_2_ Uptake (mmolg^−1^) at 25 °C and 1 bar	Refs.
^1^ N1N40G30	Anhydrous NH_3_	520	0.47	1.81	[48]
^2^ CTPC-A	PC, CO_2_, Mechanochemical	1256	0.42	3.6	[49]
^3^ MC-Serine-0.3	Zinc acetate, Mechanochemical	471	0.51	2.5	[50]
^4^ N-OMCs-0.1	KOH	566	0.31	2.43	[20]
^5^ TG-C700-4K	PO, CO_2_	1192	0.90	3.6	[26]
TFG-1	One step CO_2_	868	0.72	2.93	This work
TPG-1	One step CO_2_	888	0.63	3.2	This work
TPG-1.25	One step CO_2_	883	0.73	3.18	This work

^1^ Carbons containing N derived from chestnut tannin and glyoxal under neutral conditions. ^2^ Activated carbons derived from chestnut tannin through mechanochemical using potassium citrate (PC) as an activator. ^3^ N-doped mesoporous carbon obtained through one step mechanochemical approach using zinc acetate salt. ^4^ N-containing ordered mesoporous carbons obtained from phenol- formaldehyde resin. ^5^ Activated carbons derived from tannin and glyoxal using potassium oxalate (PO) as an activator.

## Data Availability

The data presented in this study are available upon request from the authors.

## Supplementary Materials

The following supporting information can be downloaded at: https://www.mdpi.com/article/10.3390/nano13152262/s1, Figure S1: TEM images of TPG-1 at different magnifications 20 nm (a), and 50 nm (b); Figure S2: TEM images of TFG-0.5 (a), TFG-1.25 (b), and TFG-1.5 (c); Figure S3: N2 adsorption isotherm for TFG-1* (a) with the corresponding pore size distribution (b); * refers to the second batch of TFG-1 sample; Figure S4: N2 adsorption isotherms for TFG carbon samples with tannin/polymer ratio of 0.75, and 1.1 (a), and the corresponding pore size distributions in the entire range of pores (b), mesopore range (c), and micropore range (d); Figure S5: N2 adsorption isotherms for TFG carbon samples with tannin/polymer ratio of 0.5, 1.25, and 1.5 (a), and the corresponding pore size distributions in the entire range of pores (b), micropore range (c), and mesopore range (d); Figure S6: N2 adsorption isotherms for TPG carbon samples with tannin/polymer ratio of 0.5, 1.25, and 1.5 (a), and the corresponding pore size distributions in the entire range of pores (b); Table S1: The textural properties for the reference carbon samples.
