# Supplementary material for: One-Pot Mechanochemical Synthesis of Carbons with High Microporosity and Ordered Mesopores for CO2 Uptake at Ambient Conditions
Source: Nanomaterials (Basel). 2023 Aug 7;13(15):2262. doi: 10.3390/nano13152262 (PMC10421447; doi:10.3390/nano13152262)
Supplement: Supplementary file 1 [file nanomaterials-13-02262-s001.zip › nanomaterials-2512489-supplementary.pdf]

Supplementary Materials

One-pot mechanochemical synthesis of high microporosity and ordered mesoporous carbons for CO_2 uptake at ambient conditions

Rabindra Dubadi, Mietek Jaroniec*

[1]Department of Chemistry and Biochemistry, Kent State University, Kent, OH, 44242, USA

*Correspondence: jaroniec@kent.edu

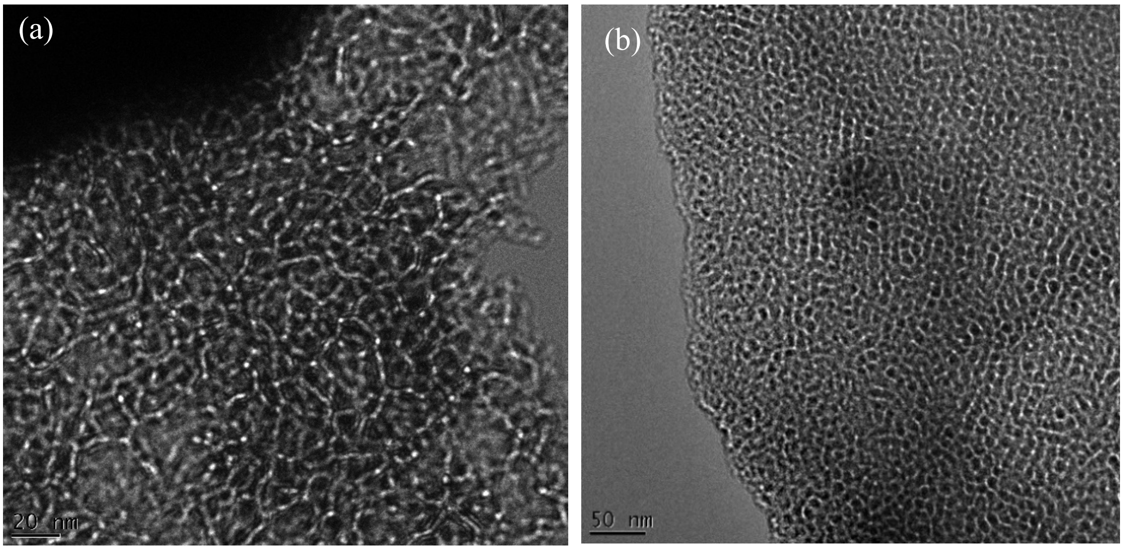

Figure S1. TEM images of TPG-1 at different magnifications 20 nm (a), and 50 nm (b).

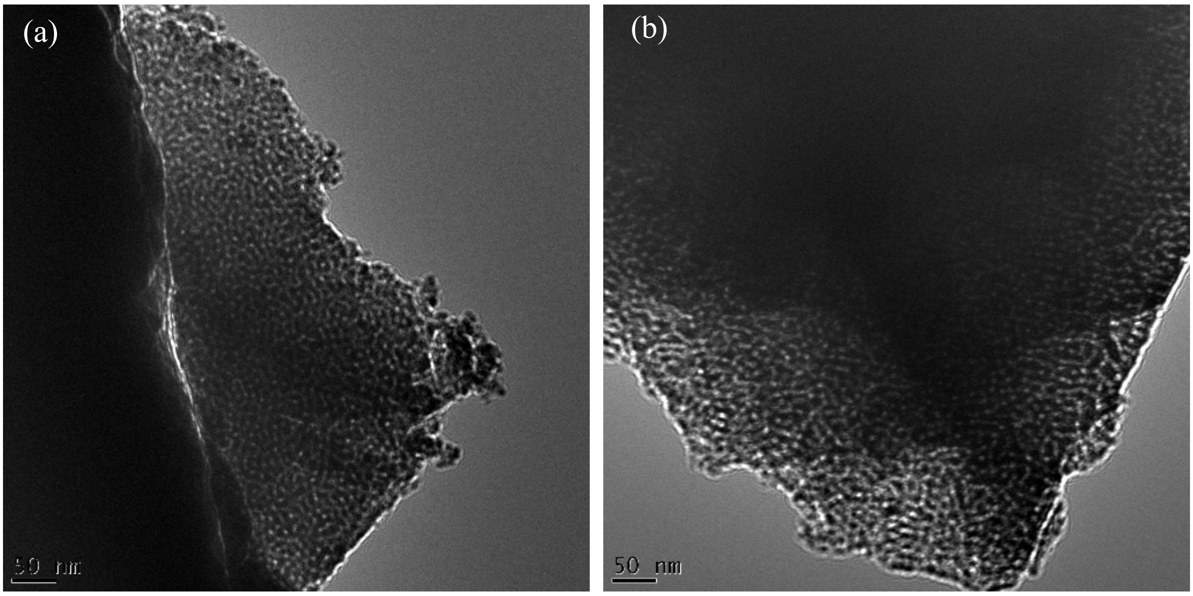

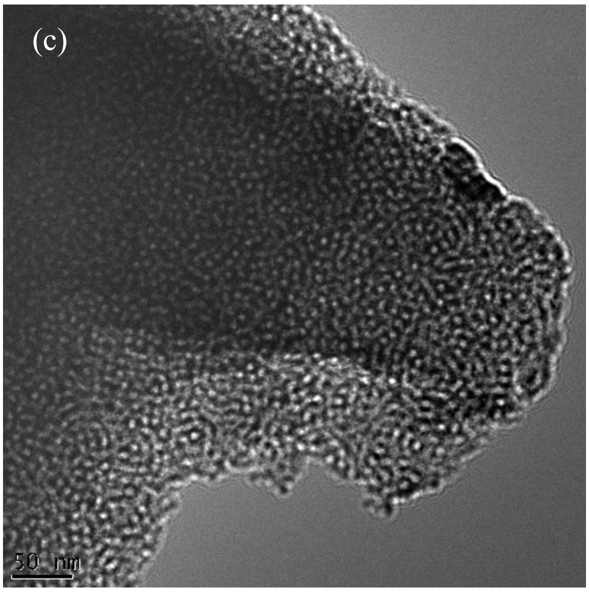

Figure S2. TEM images of TFG-0.5 (a), TFG-1.25 (b), and TFG-1.5 (c).

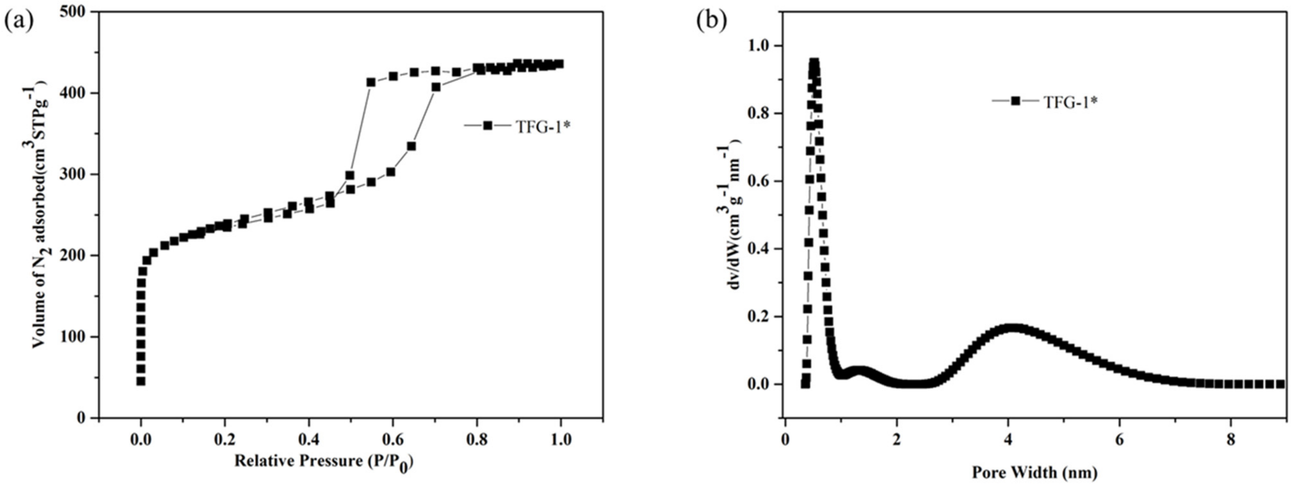

Figure S3. N_2 adsorption isotherm for TFG-1* (a) with the corresponding pore size distribution (b); * refers to the second batch of TFG-1 sample.

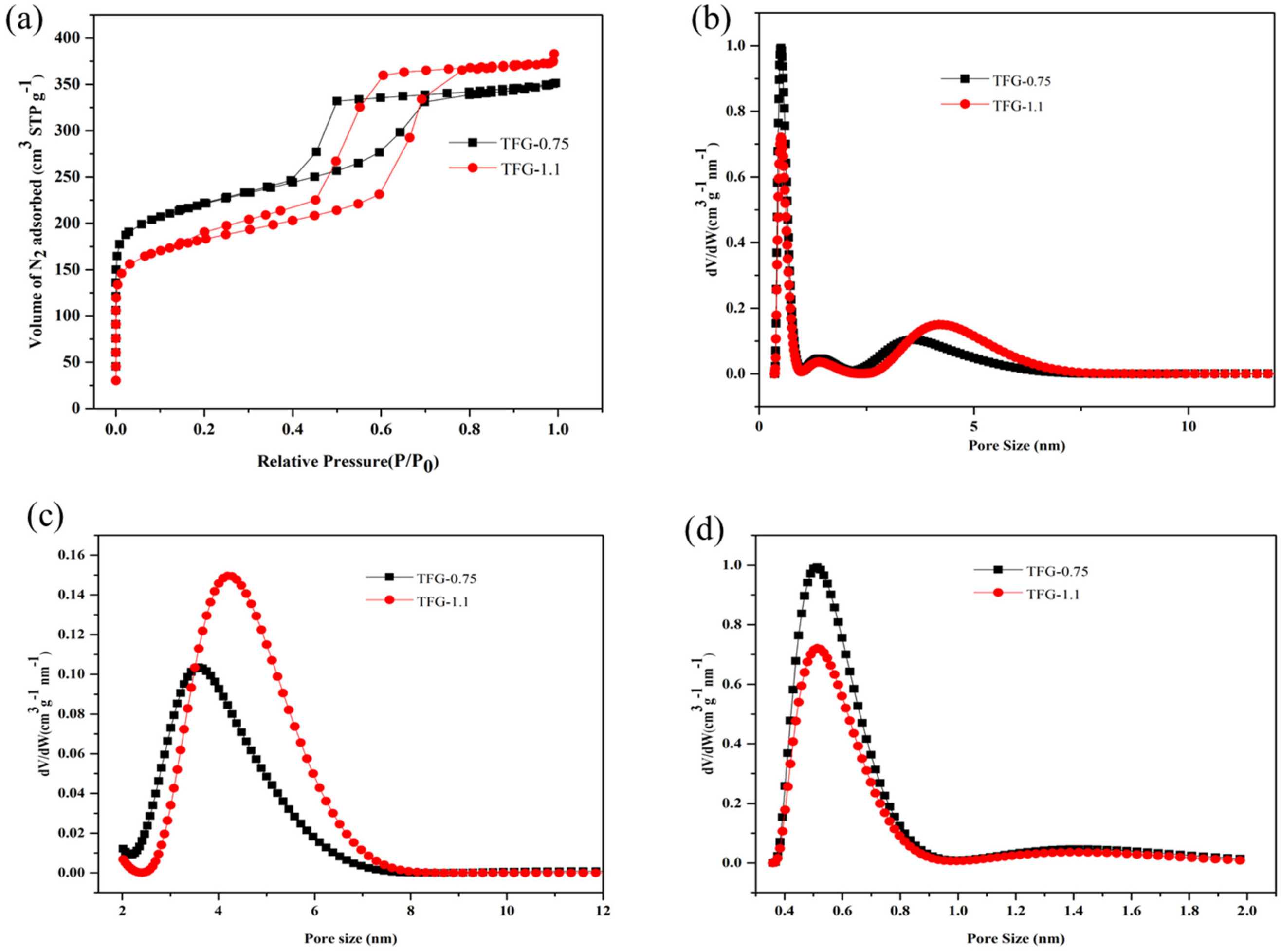

Figure S4. N_2 adsorption isotherms for TFG carbon samples with tannin/polymer ratio of 0.75, and 1.1 (a), and the corresponding pore size distributions in the entire range of pores (b), mesopore range (c), and micropore range (d).

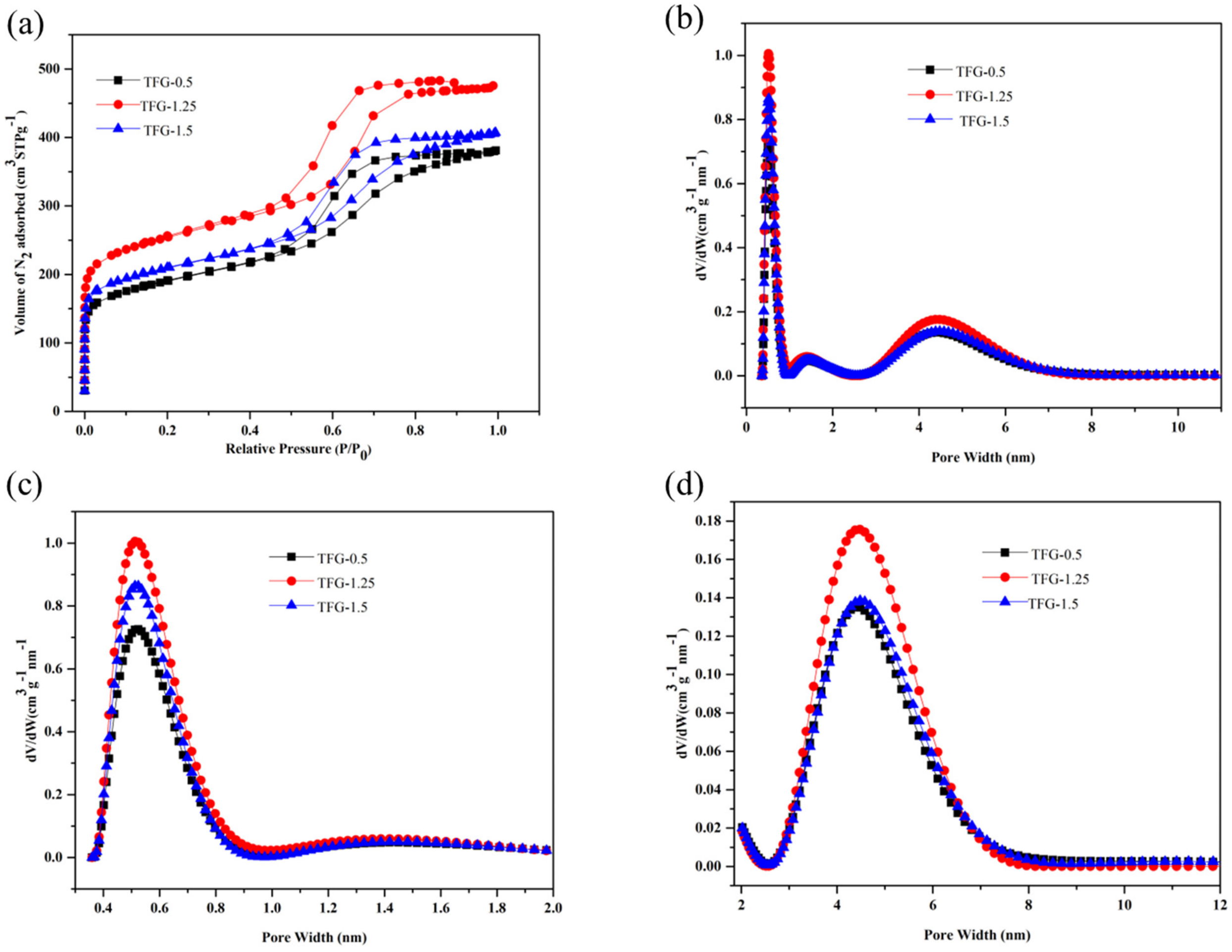

Figure S5. N_2 adsorption isotherms for TFG carbon samples with tannin/polymer ratio of 0.5, 1.25, and 1.5 (a), and the corresponding pore size distributions in the entire range of pores (b), micropore range (c), and mesopore range (d).

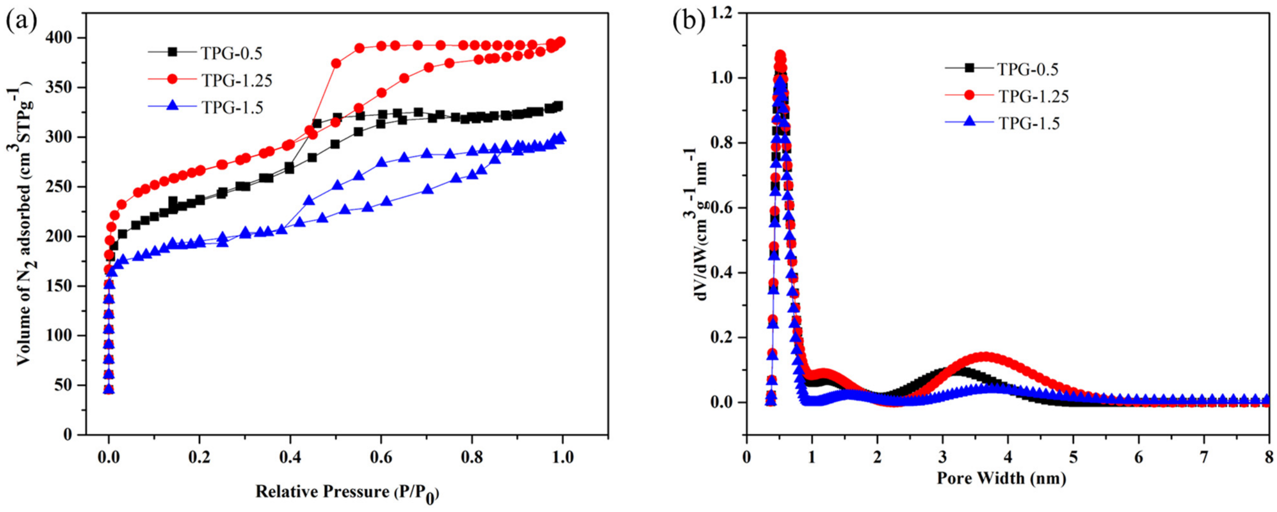

Figure S6. N_2 adsorption isotherms for TPG carbon samples with tannin/polymer ratio of 0.5, 1.25, and 1.5 (a), and the corresponding pore size distributions in the entire range of pores.

Table S1: The textural properties for the reference carbon samples.

Samples	V_{Total} (cm^3g^{-1})	$V_{<0.7nm}$ (cm^3g^{-1})	$V_{<1nm}$ (cm^3g^{-1})	$V_{<2nm}$ (cm^3g^{-1})	V_{me} (cm^3g^{-1})	Micropore width at PSD (max), w(nm)	S_{BET} (m^2g^{-1})	n_{CO2} (25 °C) ($mmolg^{-1}$)
TFG-1N	0.41	0.09	0.11	0.14	0.28	0.52	460	-
TFG-1N_2A	0.55	0.16	0.18	0.20	0.35	0.51	647	2.17
TFG-1* (2nd batch)	0.67	0.21	0.25	0.28	0.39	0.54	837	-
TPG-1N	0.38	0.07	0.09	0.13	0.25	0.51	423	2.17
TF-1	0.40	0.14	0.16	0.18	0.22	0.75	472	-
TP-1	0.46	0.20	0.23	0.26	0.20	0.77	640	-
T	0.96	0.04	0.10	0.14	0.82	2.03	450	-

Note: TFG-X and TPG-X refer to the carbon samples prepared by using Mimosa tannin with Pluronic F127 and P123, respectively, along with glyoxal as a cross linking agent. N refers to the sample carbonized in nitrogen without CO_2 activation, and N_2A refers to the sample carbonized in N_2 followed by CO_2 activation; - data not available for CO_2 capture; S_{BET} - specific surface area calculated using the BET equation for adsorption data in the relative pressure range of 0.05–0.20; Single point pore volume obtained from the volume adsorbed at 0.98 P/P_0; Pore diameter at the maximum of PSD in the micropore region was obtained by NLDFT method; n_{CO2} – amount of CO_2 adsorbed at 1.03 bar.
